# The association of breast feeding for at least six months with hemodynamic and metabolic health of women and their children aged three years: an observational cohort study

**DOI:** 10.1186/s13006-023-00571-3

**Published:** 2023-07-19

**Authors:** Maleesa M. Pathirana, Prabha H. Andraweera, Emily Aldridge, Madeline Harrison, Jade Harrison, Shalem Leemaqz, Margaret A. Arstall, Gustaaf A. Dekker, Claire T. Roberts

**Affiliations:** 1 Adelaide Medical School, The University of Adelaide, Adelaide, South Australia 5000 Australia; 2 Robinson Research Institute, The University of Adelaide, Adelaide, South Australia 5005, Australia; 3 Department of Cardiology, Lyell McEwin Hospital, Elizabeth Vale, South Australia 5112 Australia; 4 Flinders Health and Medical Research Institute, Flinders Medical Centre, Flinders University of South Australia, Bedford Park, South Australia 5042 Australia; 5 Division of Women’s Health, Lyell McEwin Hospital, Elizabeth Vale, South Australia 5112 Australia

**Keywords:** Breastfeeding, Pregnancy complications, Maternal and child health

## Abstract

**Background:**

Breastfeeding is important for both mother and child in reducing risk of future cardiovascular disease. Therefore, it may be an effective method to improve cardio-metabolic health, particularly those who are exposed to pregnancy complications which increase later CVD risk for both mother and child. The aim of this study is to assess differences in cardiometabolic health at three years postpartum in mothers who breastfed for at least six months and their children compared to those who did not.

**Methods:**

Women and children from the Screening Tests to Predict Poor Outcomes of Pregnancy (STOP) study (2015–2017) were invited to attend a health check-up at three years postpartum. Women’s breastfeeding status at least six months postpartum was ascertained through their child health record. Anthropometric and hemodynamic measurements were taken from women and their children. A fasting blood sample was taken from women to measure blood glucose and lipids.

**Results:**

A total of 160 woman-child dyads were assessed in this study. Women who breastfed for at least six months had significantly lower maternal BMI, systolic blood pressure, diastolic blood pressure, mean arterial pressure, central systolic blood pressure, and central diastolic blood pressure than those who did not and this did not change after adjusting for BMI and socioeconomic index in early pregnancy, prenatal smoking and maternal age in early pregnancy. Subgroup analysis on women who had one or more pregnancy complications during the index pregnancy (i.e. preeclampsia, gestational hypertension, delivery of a small for gestational age infant, delivery of a preterm infant, and/or gestational diabetes mellitus) demonstrated that women who breastfed for at least six months had significantly lower maternal systolic and diastolic blood pressures, serum insulin and triglycerides, and higher HDL cholesterol. There were no differences in child anthropometric or hemodynamic variables at three years of age between those children who had been breastfed for at least six months and those who had not.

**Conclusion:**

Breastfeeding for at least six months may reduce some maternal; cardiovascular risk factors in women at three years postpartum, in particular, in those who have experienced a complication of pregnancy.

**Trial registration:**

ACTRN12614000985684 (12/09/2014).

**Supplementary Information:**

The online version contains supplementary material available at 10.1186/s13006-023-00571-3.

## Background

Cardiovascular disease (CVD) is a leading cause of death among Australian women, with hospitalisation rates for females with CVD continuing to increase [1]. The challenge is to identify women at risk at an earlier age who would benefit from preventative strategies. Systematic reviews and meta-analyses indicate that women who experience major pregnancy complications such as preeclampsia and gestational diabetes mellitus, have twice the risk of developing later CVD and metabolic diseases than women who had uncomplicated pregnancies [2, 3]. Furthermore, their children are also at risk of impaired metabolic health earlier in life [4, 5]. This affords an opportunity to identify women at potential future risk and to offer early interventions to reduce or delay future CVD for themselves and their children. Women with young babies tend to engage often with the health care system allowing opportunities to provide prevention strategies to young mothers.

Breastfeeding is considered as “the gold standard for infant feeding” [6]. The World Health Organization recommends breastfeeding exclusively for up to six months [7]. It has been shown that breastfeeding for over 12 months promotes a significant reduction in both chronic hypertension and diabetes in women [8]. A systematic review and meta-analysis of breastfeeding effects on metabolic health in offspring showed that breastfeeding for any length of time provides adequate nutrition to children and decreases the risk of developing obesity and type 2 diabetes mellitus (T2DM) compared to those who are not breastfed [9]. However, this review did not adequately address confounding and results can only be interpreted with caution. A limitation of this review is that discrepancies between studies are influenced by confounding by lifestyle factors, including smoking (RR 1.76 [95% CI 1.59, 1.95]) and maternal educational status (RR 2.28 [95% CI 1.92, 2.70]), with likelihood of not initiating breastfeeding [10]. Assessment of confounding factors is therefore necessary to guide high quality evidence on the effect of breastfeeding on maternal and child metabolic health.

Evidence is clear that pregnancy complications (affecting 30% of all Australian pregnancies), such as gestational diabetes mellitus (GDM), preeclampsia, gestational hypertension, spontaneous preterm birth (sPTB) and delivery of a small-for-gestational-age infant, confer an overall twofold increased risk for later life CVD in women [11, 12]. Pregnancy complications also have long lasting implications for offspring health, likely through epigenetic changes in response to an adverse intrauterine environment [13–15]. Systematic reviews of the literature have shown that breastfeeding can improve the risk of T2DM in women with a history of GDM [16], and improve the cardio-metabolic health of offspring who were small for gestational age [17]. Therefore, it will be important to assess whether breastfeeding is beneficial for women who are at high cardiovascular risk and their offspring, which can be implemented as an interventional strategy in clinical practice.

The primary aim of this study is to assess the differences in cardiovascular and metabolic risk factors at three years in mothers who breastfed for at least six months and their children who were breastfed for at least six months, compared to those who did not. Our secondary aim is to assess the same cardiometabolic outcomes in a subgroup of women who experienced at least one pregnancy complication in their index pregnancy.

## Methods

### Study population

The study participants included women and their children from the Screening Tests to Predict Poor Outcomes of Pregnancy (STOP) study [18]. The STOP study was a prospective cohort study that aimed to assess women’s risk for pregnancy complications. A total of 1,383 nulliparous women in their first trimester were originally recruited during the period 2015–2017. Inclusion criteria for the STOP study were nulliparous women ≥ 18 years of age who were recruited at 9–16 weeks’ gestation. Women were excluded from the study if they were considered at high risk of a pregnancy complication (i.e. preeclampsia, small for gestational age delivery, spontaneous preterm birth or placental abruption) due to underlying medical conditions such as pre-existing chronic hypertension on antihypertensive medication or with a blood pressure ≥ 140/90 mmHg at 15 weeks of gestation, gynaecological history, three or more miscarriages or terminations of pregnancy, or if they received interventions that modify pregnancy outcome.

Majority of the participants were recruited from the Lyell McEwin Hospital in northern Adelaide, which services one of the most socioeconomically disadvantaged regions in metropolitan Australia. This area harbours some of the highest rates of chronic disease, diabetes, heart disease and mental illness in Australia/South Australia [19, 20]. For the STOP follow-up study, women were contacted using phone numbers provided during the STOP study, or from hospital records. If women could not physically attend an appointment, an external participation package was posted to their address and returned via paid postage. Ethics approval was granted by the Central Adelaide Local Health Network (STOP study: HREC/14/WCHN/90; STOP follow-up: HREC 18/CAHLN/318).

### Clinical data

In the original STOP study, detailed information was collected at 9–16 weeks’ (average 11 weeks’), and 34 weeks’ gestation and after delivery of the baby. Gestational hypertension was defined as systolic blood pressure ≥ 140 mmHg and/or diastolic blood pressure ≥ 90 mmHg on two or more measurements six hours apart after 20 weeks’ gestation. Preeclampsia was defined using the revised International Society for the Study of Hypertension in Pregnancy definition of gestational hypertension or postpartum hypertension with proteinuria (24-h urinary protein of 300 mg or spot urine protein/creatinine ratio of ≥ 30 mmol/L creatinine or urine dipstick protein ≥  + +) or any multisystem complication of preeclampsia or utero-placental dysfunction as evidenced by intrauterine growth restriction. Small-for-gestational-age-delivery was defined as a birth weight below the 10^th^ customized centile adjusted for maternal height, weight, parity and ethnicity, gestational age at delivery, and infant sex using the GROW centile calculator [21]. sPTB was defined as spontaneous preterm labour or preterm premature rupture of membranes resulting in a preterm birth at < 37 weeks of gestation. Gestational diabetes mellitus is screened for at 24–28 weeks’ gestation in Australia. GDM was diagnosed at 24–28 weeks’ gestation according to the International Association of Diabetes in Pregnancy Study Group (IADPSG) criteria (i.e. one or more values equal to or exceeding: fasting plasma glucose of 5.1 mmol/L, and/or a two hour plasma glucose level of 8.5 mmol/l following a 75 g Oral Glucose Tolerance Test (OGTT) [22]. Women who were at high risk of GDM completed a 75 g OGTT in their first trimester and, if normal, the OGTT was repeated at 24–28 weeks’ gestation. Data collected after delivery included newborn weight, length, arm circumference, birthweight centile, and data on complications during the neonatal period and type of feeding at discharge from hospital.

Women were recruited into the STOP follow-up study within 3 months of when their first child reached three years of age. Appointments were completed at the Clinical Trials Unit at the Lyell McEwin Hospital or completed externally as a postage paid package during the COVID-19 pandemic. Heights of women and children were measured with a stadiometer to the nearest 0.1 cm. Children’s weights were measured with a standard balance beam scale to the nearest 100 g. Body composition in women was assessed using the TANITA SC-330 bioimpedance scale (Tokyo, Japan), which measured fat to the nearest 0.1 kg, fat percentage, fat mass, fat free mass and body mass index (BMI). Those who participated in the study externally, self-reported weight and height only. Body composition in children was assessed by standardized BMI score (BMI-SDS) based on the centre for disease control (CDC) growth charts for children and teenagers aged 2 to 19 years of age [23]. Waist circumference was measured in both women and children to the nearest 0.1 cm, based on the World Health Organization guidelines [24]. Peripheral systolic and diastolic blood pressures were assessed using the USCOM BP + (USCOM, Sydney, Australia) using appropriately sized cuffs for arm circumference while participants were seated. The USCOM BP + was also used to perform a non-invasive measure of cardiovascular function, including central systolic and diastolic blood pressure, peripheral blood pressure, arterial stiffness and tone (assessed as augmentation index (AIx)), pulse rate variability and ventricular contractility (assessed as dP/dt max) [25]. Additional Table 1 highlights specified details of these variables. The USCOM BP + has been validated for use in children [26]. Cases were excluded if the signal to noise ratio, an indicator of blood pressure recording quality, was < 6. Fasting blood samples were collected from women to assess glucose, HbA1C, insulin, non-HDL lipids, HDL-cholesterol, and C-reactive protein. Insulin resistance was calculated using the Homeostatic Model Assessment for Insulin Resistance (HOMA-IR) using fasting blood glucose and fasting insulin values [27]. Some fasting blood data are missing due to some participants being pregnant or due to non-compliance. These numbers are reported in the results. Some children’s data are missing due to non-compliance and numbers are reported accordingly in the results.

**Table 1:** Demographic characteristics from the woman-child dyads who participated in the STOP three year follow-up study

Variable	Breastfed for at least six months (*n* = 74)	Did not breastfeed for at least six months (*n* = 86)	*p*-value
Socioeconomic Index (Mean (SD))	33.2 (13.7)	34.3 (15)	0.141
Caucasian ethnicity	61 (82.4%)	80 (93.0%)	0.144
**Education Status**			**0.001**
Did not complete year 10	0	1 (1.2%)	
Year 10	4 (5.4%)	12 (14.0%)	
Year 12	14 (18.9%)	25 (29.1%)	
Certificate	30 (40.5%)	33 (38.4%)	
Bachelor	23 (31.1%)	11 (12.8%)	
Higher Degree	3 (4.1%)	4 (4.7%)	
BMI at 9–16 weeks’ gestation	27.4 (7.3)	29.1 (8.0)	0.332
Pregnancy Complications^a^			
Gestational Diabetes	14 (18.9%)	14 (16.3%)	0.077
Gestational Hypertension	5 (6.8%)	7 (8.1%)	0.741
Preeclampsia	6 (8.1%)	9 (10.5%)	0.610
Small for gestational age baby	12 (16.2%)	9 (10.5%)	0.283
Spontaneous preterm birth	4 (5.4%)	3 (3.5%)	0.554
Gestational age (weeks) (Mean (SD))	39.5 (1.7)	39.4 (1.7)	0.754
Child birthweight (g) (Mean (SD))	3265.3 (491.2)	3360 (531.9)	0.612
Current Maternal Age (Mean (SD))	31.8 (5.0)	31.4 (5.2)	0.655

^a^Pregnancy complications are not mutually exclusive and individual women can have multiple pregnancy complications

### Breastfeeding status

Duration of breastfeeding was ascertained by collecting information on breastfeeding at 1–4 weeks, 6–8 weeks, 6–9 months, and 18–24 months of age from the child’s “blue book” (i.e. Child Health record) which is given to all parents of newborns in South Australia. This data is collected by a child health nurse or their general practitioner who record the self-report of the mother at the time of assessment. To tick yes for breastfeeding at certain timepoints, a woman had to have been exclusively breastfeeding or in combination with other feeding (either with formula or solids).

### Statistical analysis

Data were analysed using IBM SPSS Version 26. Women who breastfed for at least six months were compared to those who did not. Similarly, children who had been breastfed for at least six months were compared to those who had not. The justification to select this time point for breastfeeding status is based on the World Health Organization recommendation that children should be exclusively breastfed up until six months of age.

Subgroup analysis was undertaken assessing women who experienced a complicated index pregnancy (i.e. diagnosis of one or more of the following: preeclampsia, gestational hypertension, GDM, delivery of a small for gestational age infant, delivery of a preterm infant, spontaneous PTB). Bivariate analysis was used to compare anthropometric and hemodynamic variables between the two groups with data presented as mean (SD), n (%) or median (IQR). Effect sizes were reported for primary outcomes (mean difference; MD (95% CI)). To address the concern of confounding, significant associations between breastfeeding/being breastfed for at least six months postpartum and maternal or child metabolic risk factors were analysed using linear regression. Model one was adjusted for BMI and socioeconomic index (SEI), which was defined by the New Zealand Socioeconomic Index (NZSEI) at index pregnancy. SEI is scored between a value of 10 to 90; with a lower score reflecting greater socioeconomic disadvantage [28]. Model two was adjusted for maternal age in early pregnancy and prenatal smoking (i.e. smoking in first trimester, yes or no). These variables were selected as they are known to be associated with both cardio-metabolic health and breastfeeding duration.

## Results

**Fig. 1:**
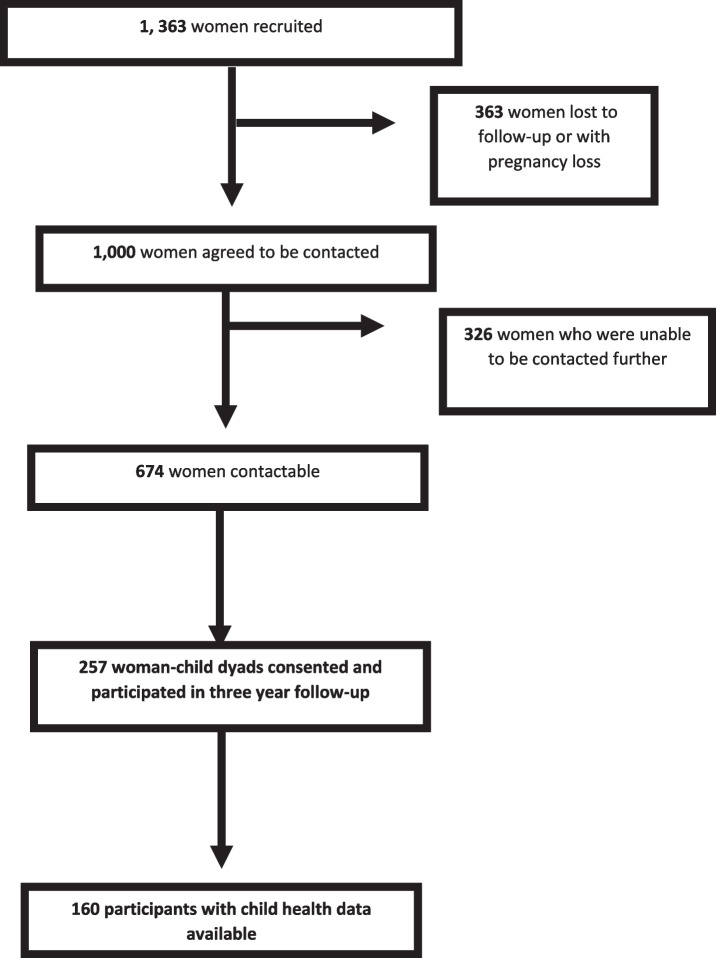
Flow chart of follow-up participant selection for the STOP three year follow-up study

A total of 1,383 women were recruited to the STOP pregnancy study. Figure 1 demonstrates the flow chart of participant selection. Of these women, 1,000 agreed to be contacted for future studies at the time of their index pregnancy. However, only 674 were contactable at the time of follow-up. Of these, 257 woman-child dyads consented and participated in the follow-up study from January 2019 until June 2021. Of these participants, 160 women had adequate child health data with information on breastfeeding, therefore data for these participants were analysed in this study (Fig. 1). Of these women, 12 completed the study externally such that anthropometric and hemodynamic data are incomplete. One hundred and sixty women reported breastfeeding at 1-4 weeks postpartum (100%), 130 women reported breastfeeding at 6-8 weeks postpartum (76.5%), seventy women (46.9%), reported breastfeeding at 6-9 months postpartum and 13 women (8.1%) reported breastfeeding at 12-18 months postpartum. Additional Table 1 shows attrition analysis for patients who did and did not attend the follow-up study, patients who had recorded breastfeeding status at 6–9 months, and patients who did and did not complete pathological blood testing. BMI was significantly higher in those who were followed up compared to those who were not (28 ± 7.2 vs. 27.9 ± 7.1 *p* = 0.020), more women were white (n = 246 (88.8%) vs. 888 (81%) *p* = 0.000). More women who attended follow up were university educated (51 (18.4%) vs. 154 (14.1%) *p* = 0.000). Of the women who completed their blue book data, as well as the women who complied with postpartum blood testing and physiological measurements, more of these participants had bachelor’s degrees than those who did not (*n* = 34 (21.3%) vs. 20 (15.9%) *p* = 0.017) (*n* = 10 (9.7%) vs. 10 (5.3%)) *p* = 0.024), respectively.

Demographic characteristics of the participants who attended the three year follow-up are presented in Table 1. There were no differences in baseline parameters such as maternal age, SEI, BMI in first trimester, child birthweight, gestational age at delivery, and waist circumference at three years postpartum between women who breastfed for at least six months and those who did not. Educational status at baseline was significantly different between those who breastfed and those who did not (Bachelor’s Degree Completed 23 (31.1%) vs. 11 (12.8%) (*p* = 0.001). Those who attended the follow-up study in their index pregnancy had significantly higher SEI than those who did not attend (37.1 ± 16.8 vs. 33.4 ± 12.5 *p* = 0.001*,* on a scale of 10–90) (Additional Table 2).

**Table 2:** Cardiometabolic risk factors at three years postpartum in women and offspring at age 3

*Anthropometric and hemodynamic variables at three years*			
	Breastfed for at least six months (*n* = 70)	Did not breastfeed for at least six months (*n* = 74)	Effect Size (MD, 95% CI)^
BMI (kg/m2)	28.4 (8)	31.6 (10.2)	**-3.34 (6.5, -0.2)**
Fat mass (kg)	29.1 (17.3)	36.4 (19.3)	1.2 (-3.7, 6.1)
Waist circumference (cm)	88.6 (20.6)	95.9 (20.5)	**-9.1 (-17.8, -0.2)**
Systolic blood pressure (mmHg)	119.0 (12.7)	122.5 (14.6)	-**6.5 (-10.8, -2.2)**
Diastolic blood pressure (mmHg)	66.9 (9.3)	70.2 (11.3)	**-5.1 (-8.3, -1.7)**
Mean arterial pressure (mmHg)	80.8 (9.8)	85.1 (14.1)	**-5.2 (-9.1, -1.1)**
Augmentation Index (%)	53.1 (18)	56.7 (22.8)	3.4 (-4.3, 9.1)
Central systolic blood pressure (mmHg)	109.4 (11.9)	111.9 (13.9)	**-4.9 (-9.1, -0.9)**
Central diastolic blood pressure (mmHg)	69.6 (9.4)	73.1 (11.8)	**-5.4 (-8.7, -2.0)**
*Fasting blood values*			
	Breastfed for at least six months (*n* = 24)	Did not breastfeed for at least six months (*n* = 30)	Effect Size (MD, 95% CI)^
Fasting glucose (mmol/L)	4.7 (0.5)	4.7 (0.5)	-0.163 (-0.4, 0.1)
Insulin(mU/L)*	8.1 (3.1)	13.2 (9.2)	-3.9 (-9.0, 1.2)
HOMA-IR*	1.7 (0.6)	2.8 (2.1)	-0.04 (-0.2, 0.19)
Triglycerides (mmol/L)**	0.9 (0.4)	1.3 (7.5)	**-0.57 (-0.95, -0.19)**
HDL-C (mmol/L)	1.4 (0.8)	1.4 (0.4)	**0.202 (0.007, 0.396)**
LDL-C (mmol/L)	1.2 (0.3)	2.6 (0.7)	0.03 (-0.36, 0.43)
Total Cholesterol/HDL ratio	3.2 (0.8)	4.8 (5.3)	-0.07 (-2.4, 2.3)
Non-HDL Cholesterol	3.1 (0.8)	3.2 (0.8)	-0.19 (-0.64, 0.26)
Total Cholesterol (mmol/L)	4.3 (0.9)	4.4 (0.8)	0.04 (-0.48, 0.55)
C-Reactive Protein	3.8 (4.8)	3.4 (2.5)	0.67 (-1.5, 2.9)
	Offspring who were breastfed for at least six months (*n* = 68)	Those who were not breastfed for at least six months (*n* = 71)	Effect Size (MD, 95% CI)^
BMI SDS^	55.6 (29.8)	58.4 (31.7)	-1.7 (-9.1, 12.6)
Waist circumference*	51.1 (3.5)	51.8 (4.5)	-0.6 (-2.1, 0.8)
^^	**(*n* = 38)**	**(*n* = 34)**	
Systolic blood pressure (mmHg)	101.9 (12.1)	96.1 (18.5)	6.8 (-1.3, 15)
Diastolic blood pressure (mmHg)	58.8 (10.5)	58.1 (15.8)	1.3 (-5.7, 8.3)
Mean arterial pressure (mmHg)	73.1 (13.3)	71.1 (18.3)	4.2 (-4.1, 12.6)
Augmentation Index (%)	87.2 (38.4)	98.3 (52.5)	5.4 (-9.1, 19.9)
Central systolic blood pressure (mmHg)	94.2 (12.4)	91.6 (20.3)	2.0 (-5.6, 9.7)
Central diastolic blood pressure (mmHg)	62.6 (10.1)	61.2 (13.0)	2.3 (-4, 8.7)

Bold indicates significant effect size

The reduced sample size for maternal blood tests was due to noncompliance of participants who did not complete respective blood tests

^*^P-value = 0.001 for univariate analysis

^**^P-value = 0.006 for univariate analysis

^based on univariate linear regression

^BMI SDS is adjusted for age and sex [23], all other outcomes are adjusted for child age

^^The sample size for child hemodynamic variables is smaller due to noncompliance with the USCOM BP +

## Association of breastfeeding for at least six months with cardiometabolic outcomes in women and children

At three years postpartum, BMI was 3.34 kg/m^2^ lower in those who breastfed for at least six months compared to those who did not (95% CI -6.5, -0.2), and waist circumference was lower in those who breastfed compared to those who did not (MD -9.1 (95% CI -17.8, -0.2)). For hemodynamic variables, systolic blood pressure (MD -6.5 mmHg (95% CI -10.8, -2.2)), diastolic blood pressure (MD -5.1 mmHg (95% CI -8.3, -1.7)), mean arterial pressure (MD -5.2 mmHg (95% CI -9.5, -1.1)), central systolic blood pressure (-4.9 mmHg ( 95% CI -9.1, -0.9)), and central diastolic blood pressure (MD -5.4 mmHg (95% CI -8.7, -2.0)) were all lower in women who breastfed for at least six months compared to those who did not. These did not change after adjusting for covariates (Table 3). Data for fasting blood samples were available for 54 women (Table 2). Serum triglycerides were lower in those who breastfed for at least six months (MD -0.57 mmol/L 95% CI -0.95, -0.19), serum HDL-C was 0.2 mmol/L higher in women who breastfed for at least six months (MD 0.2 mmol/L (95% CI 0.007, 0.396). This association was attenuated by BMI and SEI in first trimester but not attenuated by prenatal smoking and age in first trimester (Table 3). No differences were seen for anthropometric and hemodynamic variables between offspring who were breastfed for at least six months compared to those who were not (Table 2).

**Table 3:** Mean differences in maternal cardiovascular risk factors at three years postpartum assessed by linear regression

	Model 1^a^ Adjusted Mean Difference (95% CI)	Model 2^b^ Adjusted Mean Difference (95% CI)
BMI	-1.7 (-3.6, 0.14)	-3.3 (-6.4, -0.17)
Waist circumference (cm)	-4.9 (-10.8 to 1)	-7.7 (-16.5, -1.1)
Systolic blood pressure (mmHg)	**-5.7 (-9.7**, **-1.8)**	**-6.4 (-10.6**, **-2.1)**
Diastolic blood pressure (mmHg)	**-4.8 (-7.9**, **-1.7)**	**-5.0 (-8.3 to -1.7)**
Mean arterial pressure (mmHg)	**-4.5 (-8.2**, **-0.8)**	**-5.1 (-9.0**, **-1.1)**
Central systolic blood pressure (mmHg)	**-4.4 (-8.2**, **-0.5)**	**-4.8 (-8.9**, **-0.7)**
Central diastolic blood pressure (mmHg)	**-5 (-8.2**, **-1.8)**	**-5.3 (-8.6**, **-1.9)**
Insulin (mg/dL)	-1.7 (-4.6, 1.2)	-3.7 (-8.7, 1.3)
HOMA-IR^	-1.4 (-7.7, 4.8)	-0.037 (-0.26, 0.19)
Serum Triglycerides (mmol/L)	-0.6 (-1.0, -0.28)	**-0.6 (-0.9**, **-0.2)**
HDL (mmol/L)	0.2 (0.11, 0.4)	**0.20 (0.11**, **0.4)**

^a^adjusted for BMI at first trimester SEI at first trimester

^b^adjusted for prenatal smoking (first trimester) and age at first trimester

## Subgroup analysis

When stratifying for women who experienced at least one pregnancy complication, those who breastfed for at least six months had significantly lower peripheral systolic (MD -8 mmHg (95% CI -14.1, -2) and diastolic blood pressures (MD -7.6 mmHg (95% CI -12, -3.1), and lower central systolic (SD -7.5 mmHg (95% CI -13.3, -1.6) and diastolic blood pressures (SD -7.1 mmHg (95% CI -11.6, -2.5)) compared to women with at least one pregnancy complication who did not breastfed for at least six months. Women with at least one pregnancy complication at index pregnancy who breastfed for at least six months also showed lower serum insulin (MD -8.2 mmol/L (95% CI -15.0, -1.2)), triglycerides (MD -0.61 mmol/L (95% CI -1.1, -0.44)) and higher HDL-C (MD 0.29 mmol/L (95% CI 0.03, 0.5) (Table 4).

**Table 4:** Subgroup analysis of women and children exposed to complicated pregnancies

	Women with complicated pregnancies who breastfed for at least six months (*n* = 34)	Women with complicated pregnancies who did not breastfeed for at least six months (*n* = 35)	Effect Size (MD, 95% CI)^
*Anthropometric and hemodynamic variables*			
BMI (kg/m2)	28.4 (7.8)	33.0 (8.9)	-4.1 (-8.4, 3.6)
Fat mass (kg)	27.6 (16.3)	40.2 (20.3)	-9.2 (-18.8, 0.4)
Waist circumference (cm)	89.2 (21.5)	102.8 (23.4)	12.6 (-25.3, 0.1)
Systolic blood pressure (mmHg)	121.2 (12.1)	124.5 (15.6)	**-8.0 (-14.1**, **-2)**
Diastolic blood pressure (mmHg)	67.7 (9.5)	72.5 (11.1)	**-7.6 (-12.0**, **-3.1)**
Mean arterial pressure (mmHg)	81.6 (9.8)	88.3 (14.5)	-8.3 (-13.3, 2.9)
Augmentation Index (%)	52.2 (16.6)	61 (22.1)	-1.6 (-11.2, 8.0)
Central systolic blood pressure (mmHg)	111.2 (12)	115.4 (14.7)	**-7.5 (-13.3**, **-1.6)**
Central diastolic blood pressure (mmHg)	70.9 (9.5)	75.3 (11.4)	**-7.1 (-11.6**, **-2.5)**
*Pathology results*			
	Women with complicated pregnancies who breastfed for at least six months (*n* = 13)	Women with complicated pregnancies who did not breastfeed for at least six months (*n* = 19)	Effect Size (MD, 95% CI)^
Fasting glucose (mmol/L)	4.8 (0.5)	4.7 (0.5)	-0.155 (-0.5, 0.19)
Insulin(mU/L)*	7.5 (2.1)	16.5 (10.2)	**-8.2 (-15.0**, **-1.2)**
HOMA-IR*^	1.6 (0.5)	3.5 (2.2)	-0.17 (-0.5, 0.15)
Triglycerides (mmol/L)**	1.0 (0.5)	1.6 (0.8)	**-0.61 (-1.1**, **-0.44)**
HDL-C (mmol/L)	1.3 (0.3)	1.2 (0.3)	**0.29 (0.03**, **0.5)**
LDL-C (mmol/L)	2.7 (0.5)	2.7 (0.5)	0.28 (-0.11, 0.66)
Total Cholesterol/HDL ratio	3.4 (0.8)	6.2 (7.2)	2.1 (-3.7, 4.6)
Non-HDL Cholesterol	3.1 (0.6)	2.5 (0.6)	0.03 (-0.4, 0.5)
Total Cholesterol (mmol/L)	4.4 (0.5)	4.6 (0.6)	0.29 (-0.3, 0.8)
C-Reactive Protein	3.0 (4)	3.8 (2.5)	-0.19 (-2.7, 2.4)
	Offspring exposed to pregnancy complication(s) in utero that were breastfed for at least six months (*n* = 30)	Offspring exposed to uncomplicated pregnancy in utero that were breastfed for at least six months (*n* = 9)	Effect Size (MD, 95% CI)^
BMI SDS^^	58.8 (27.3)	56.2 (32)	-2.3 (16.9, 12.3)
Waist circumference	52 (2.7)	50.9 (2.8)	-0.5 (-3.0, 1.9)
Systolic blood pressure (mmHg)	99.6 (11.0)	91.9 (24.6)	10.2 (-3.2, 23.7)
Diastolic blood pressure (mmHg)	58.7 (11.8)	55.8 (17.5)	6.6 (-3.8, 17.1)
Mean arterial pressure (mmHg)	73.1 (14.9)	67.2 (18.8)	8.4 (-3.7, 20.6)
Augmentation Index (%)	97.8 (45.9)	81.9 (28.7)	15.1 (-13.5, 43.6)
Central systolic blood pressure (mmHg)	91.2 (13.1)	85.2 (21.2)	4.9 (-8.2, 18.1)
Central diastolic blood pressure (mmHg)	62.1 (11.8)	60 (17.8)	4.7 (-6.2, 15.8)

*P-value = 0.001 for univariate analysis

**P-value = 0.04 for univariate analysis

^^BMI SDS is adjusted for age and sex [23], all other outcomes are adjusted for child age

## Discussion

The primary aim of this observational study was to assess whether there were differences in cardiometabolic health at three years in mothers who breastfed for at least six months and their children compared to those who did not. There was a reduction in hemodynamic variables, such as peripheral blood pressure, even with adjustment for covariates. There was a reduction in anthropometric measurements, and a reduction in serum triglycerides and an increase in HDL-C but these were attenuated for covariates including BMI in first trimester and SEI. There was no difference in cardio-metabolic outcomes at three years of age between children who were breastfed for at least six months and those who were not.

Our secondary aim was to determine whether there was a difference in cardiometabolic risk factors in women exposed to at least one pregnancy complication and their children. This is important because these women and children are particularly at risk of future T2DM and CVD. Subgroup analysis of women with at least one pregnancy complication during the index pregnancy revealed that systolic and diastolic blood pressure, central systolic and diastolic blood pressure, serum insulin and triglycerides were significantly lower in those who did breastfeed for at least six months postpartum.

Maternal cardiovascular health is being brought to the forefront of clinical care. In Australia, CVD caused more female deaths than any other disease groups, (29% of deaths in 2016) including breast cancer. Cardiovascular risk calculators, such as Framingham, are not appropriate for young women [1]. However, some young women are known to exhibit risk factors of metabolic syndrome (i.e. a dangerous cluster of risk factors that contributes to heart disease). Therefore, interventions that can reduce these risk factors are necessary to reduce long term CVD risk. Our study found that there was a reduction in hemodynamic variables such as peripheral systolic and diastolic blood pressure, mean arterial pressure, and central systolic and diastolic blood pressure among women who breastfed for at least six months postpartum, even after adjusting for covariates. A study of women aged 40–65 years who reported whether they ever breastfed found that breastfeeding for at least 5 months showed a 50% reduction in coronary artery disease, however this was partially confounded by traditional risk factors [29]. Zhang et al*.* showed that not breastfeeding was associated with an increased risk of developing hypertension greater than 20 years postpartum (OR 1.18 95% CI 1.05, 1.32) including adjustment for the same confounders assessed in our study such as age, BMI and smoking [30]. The association between breastfeeding and reduced hypertension is thought to be mediated by oxytocin which is released during feeding [31]. Our finding that women who breastfed for at least six months had a reduction in central systolic and diastolic blood pressures indicates that breastfeeding may benefit vasculature and heart health, as central blood pressure reflects aortic pressure and is strongly associated with vascular disease outcomes [32].

Furthermore, our study showed that women who experienced at least one pregnancy complication who did breastfeed for at least six months had reduced peripheral and central blood pressures than the women who did not breastfeed for at least six months. Preeclampsia, which is characterized by high blood pressure during pregnancy, places women at a fourfold risk of heart failure and twofold risk of coronary heart disease [11]. These women are more likely to be hypertensive in the postpartum period [11]. Countouris et al. were able to show that lactation reduced systolic and diastolic blood pressures in the postpartum period in women with gestational hypertension (*p* = 0.02, *p* = 0.02 respectively) but not those with preeclampsia [33]. In our subgroup analysis, only 30 women had a hypertensive disorder of pregnancy (i.e. preeclampsia or gestational hypertension). Therefore, further investigation into whether breastfeeding can reduce hypertension in preeclamptic women is warranted.

Our study showed that women who breastfed for at least six months had reduced serum triglycerides and elevated HDL-C than those who did not breastfeed at three years postpartum. Many studies have shown a dose–response between lactation length and reduction in metabolic syndrome risk. Suliga et al*.* found that breastfeeding for greater than 12 months was associated with a reduction in metabolic syndrome risk (OR 0.76 95% CI 0.60, 0.95) [34]. One study by Natland et al. were able to show that women who breastfed over the course of two years had significantly lower serum triglycerides than women who had never breastfed [35]. We did not look at metabolic syndrome due to the low number of participants and low compliance with blood sampling but it is likely that our cohort has high rates of metabolic syndrome based on literature of women serviced from this demographic [36, 37]. Therefore, the improvement in serum lipid profile seen in the women who breastfed for at least six months may be important to investigate in this cohort in future.

The changes in serum lipids were also seen in our subgroup of women with at least one pregnancy complication. In our subgroup, women who had breastfed for at least six months after at least one pregnancy complication also had reduced serum insulin compared to those who did not. A study by Blair et al. found that among those with a history of GDM, breastfeeding for as little as 8 weeks had a reduced risk of hypertriglyceridemia (i.e. serum triglyceride ≥ 150 mg/dL) than those who were not breastfeeding at the same time point (aOR = 0.26 (95% CI: 0.10, 0.66), *p* = 0.005) [38]. The prevalence of metabolic syndrome in the women who breastfed was significantly lower (17.9%) compared to those who were not at 8 weeks postpartum (42.9%) (*p* < 0.001) [38]. Yu et al*.* similarly found a dose dependent response of lactation frequency with reduced risk of metabolic syndrome in a cohort of women with major pregnancy complications (adjusted OR 0.89 95% CI 0.79, 0.99) [39] The women who had pregnancy complications who breastfed for at least six months compared to the women who did not also had statistically significantly higher HDL (*p* < 0.001), lower triglycerides (*p* < 0.001), reduced systolic blood pressure (*p* = 0.001) compared to those who did not. While we had a smaller sample size for our analysis, our findings are in parallel with those reported by Yu et al*.*

Our previous systematic review and meta-analysis on breastfeeding after a GDM pregnancy did not show a difference in serum insulin between women with a history of GDM who breastfed compared to those who did not but there was a reduction in the risk of developing T2DM later [16]. Women who are diagnosed with pregnancy complications such as preeclampsia and GDM are generally more likely to be insulin resistant in the postpartum period compared to those with an uncomplicated pregnancy [40, 41]. Therefore, the evidence based on previous literature and this study suggests that encouraging breastfeeding could be beneficial to address insulin levels in women postpartum.

There were many strengths in this observational study. We were able to assess non-conventional markers of cardiovascular risk in women and children such as augmentation index and mean arterial pressure which reflect vascular health. Our cohort was recruited from a hospital servicing a low SES population enabling our findings, if replicated in larger studies, to be generalizable to disadvantaged communities. Due to the difficulties in recruiting disadvantaged participants in research, many studies report on participants in moderate to high SES communities who generally tend to have fewer cardio-metabolic risk factors and better health.

There are limitations to address in this study. Due to the low SES community, it was difficult to recruit and maintain engagement in the cohort with a high percentage unable to be contacted for follow-up. Just one quarter of participants from the original STOP study attended the 3 year follow-up, albeit 50% of those who consented to follow-up and were contactable. Given the low percentage of women who were retained in the follow-up study, larger cohort studies are required and the results of this study should be interpreted with caution. The women recruited into the STOP study were from a population with severe disadvantage, where engagement in exercise is much lower than the national average and the rate of diabetes is 22% higher than the national average [42]. Therefore, finding an association between breastfeeding and metabolic risk factors in this cohort may be confounded by the poorer health in the local population compared to state and national averages. It is known that low socioeconomic status has a significant impact on breastfeeding practices, which therefore may have a significant effect on our results [43]. It may be important to target women living with socioeconomic disadvantage further and provide them with more support to breastfeed their babies.

It is possible that the women who participated in our follow-up may have had cardio-metabolic risk factors pre-pregnancy. Of the women who attended the 3 year follow-up, 22% of these women had metabolic syndrome in pregnancy [44]. However, it is likely that assessing covariates from early pregnancy such as BMI, smoking status and SEI likely also reflect pre-pregnancy status and this could be accounted for in part [45, 46]. Furthermore, our pregnancy cohort consisted of nulliparous young women with no prior medical complications. However, future studies should assess women’s health status in preconception to better ascertain how this influences future breastfeeding status and metabolic health during gestation and early postpartum.

When assessing attrition, amongst the women who attended the follow-up study, those who attended had higher BMI and therefore may have greater cardiometabolic risk factors which should be considered when interpreting the results. Amongst the women who attended the follow-up study, those who had complete breastfeeding data and those who completed a blood test were more likely to have Bachelor degrees. Therefore, these women were more educated and perhaps more able to participate in follow-up. There is a degree of selection bias present as the follow-up cohort appear to be generally ‘healthier’. However, if more disadvantaged women had participated at follow-up we would expect to see a greater difference between cardiometabolic outcomes in those who did and did not breastfeed for at least six months.

As the study is observational in nature, there are variables that we cannot fully control for. Some women were pregnant at the time of follow-up so they were excluded from these analyses as anthropometric and hemodynamic variables are not comparable between pregnant and non-pregnant states. Due to the low sample size, we were unable to undertake sensitivity analyses to ascertain whether there was weighting of results based on pregnancy complications nor assess these complications individually. The current sample size is underpowered to assess hemodynamic variables based on post-hoc analysis (42%). The child health data recorded in the child’s blue book (health record) were incomplete for a significant number of women. This is because blue book completion is not mandatory. Many women take their children for check-ups to their general practitioner rather than a child nurse who would normally enter data in the book. As mentioned in the methods, breastfeeding was recorded as ‘yes’ if the women reported that she was breastfeeding in any capacity, including mixed feeding. There may be heterogeneity between women in the breastfeeding group as some may have been exclusively breastfeeding and some may have been mixed feeding and therefore affecting the findings of this paper.

Other studies undertook detailed questionnaires on lactation via telephone or in person at the time of infant follow-up, which detailed frequency of lactation and specified any addition of formula or solid foods [47]. These would provide a better profile of breastfeeding status. Although we recruited 257 participants, there were only blue book data available for 160, of whom only 54 women presented for fasting blood sampling. Furthermore, there were adequate hemodynamic data for just 72 children. Therefore, future studies will require a larger sample size.

## Conclusion

Those who breastfed for at least six months postpartum had reduced blood pressure and improved lipid profile at three years postpartum compared to those who did not, even after adjustment for covariates. A similar association was seen for women who experienced at least one major pregnancy complication at index pregnancy. It may be beneficial to provide interventions that support breastfeeding in disadvantaged women with pregnancy complications to reduce their risk of CVD in the future but further research with a larger sample size and better ascertainment of breastfeeding status is required. We were unable to show an association between hemodynamic and anthropometric parameters in offspring who were breastfed for at least six months compared to those who were not. This too requires replication in a larger sample size to confirm this association.

## Supplementary Information

**Additional file 1.**

## Data Availability

Upon request.

## Abbreviations

CVD: Cardiovascular disease
BMI SDS: Standardized BMI
GDM: Gestational Diabetes Mellitus
SEI: Socioeconomic index
STOP: Screening Tests to Predict Poor Outcomes of Pregnancy Study
T2DM: Type 2 Diabetes Mellitus
