# Supplementary material for: The association of breast feeding for at least six months with hemodynamic and metabolic health of women and their children aged three years: an observational cohort study
Source: Int Breastfeed J. 2023 Jul 19;18:35. doi: 10.1186/s13006-023-00571-3 (PMC10357869; doi:10.1186/s13006-023-00571-3)
Supplement: Supplementary file 1 — Additional file 1. [file 13006_2023_571_MOESM1_ESM.docx]

| USCOM BP+ Variable | Definition |
| --- | --- |
| Augmentation Index (Aix) | Aix is a measurement of wave reflection, which is considered a surrogate marker of arterial stiffness. It is reported as a ratio of augmentation pressure to pulse pressure (Aix = cAP/cPP) (*Heusenvield et al. 2019)* |
| Mean Arterial Pressure (mmHg) | The average arterial pressure throughout one cardiac cycle (both systole and diastole). MAP is calculated by DBP + 1/3(SP-DP)  Clinically, a MAP of 60mmHg to 100mmHg is considered “normal”.  *(DeDemers et al. 2021)* |
| Central Blood Pressure (systolic and diastolic) (mmHg) | Pressure in the ascending aorta.  Values are assessed the same as for peripheral blood pressure.  *(McEirney et al. 2014)* |

Supplementary Table 1: USCOM BP+ Variable definitions

Supplementary Table 2: Sample characteristics of full STOP cohort, those who were followed-up at 3 years postpartum and those who were not, and those who complied with blood and physiological testing and those who did not.

| **STOP study** | **Total Cohort (n= 1,300)** | **Followed-Up (n=277)** | **Not Followed-Up (n=1017)** | **p-value*** |
| --- | --- | --- | --- | --- |
| Age at recruitment | 26 (5.1) | 25.7 (5) | 27.1 (5.1) | 0.807 |
| BMI | 28 (7.2) | 28 (7.2) | 27.9 (7.1) | **0.020** |
| SEI | 32 (13.6) | 33.8 (14.2) | 32.7 (13.9) | 0.172 |
| White ethnicity | 1134 (82.5%) | 246 (88.8%) | 888 (81%) | **0.000** |
| Education Status  Did not complete  Year 10 Year 12  Certificate  Bachelor  Higher degree | 29 (2.1) 261 (19)  306 (22.3) 488 (35.5) 205 (15)  80 (5.8) | 5 (1.8%) 32 (11.6%) 32 (11.6%) 51 (36.8%) 51 (18.4%) 19 (6.9%) | 24 (2.2%) 229 (20.9%) 229 (20.9%) 286 (35.2%) 154 (14.1%) 61 (5.6%) | **0.000** |
| Pregnancy complication^ GDM Gestational hypertension Preeclampsia Preterm Birth Small for gestational age | 199 88  121 62 153 | 39 (14.1%) 18 (6.5%)  28 (10.1%) 14 (5.1%) 37 (13.4%) | 160 (15.7%) 70 (6.9%)  93 (9.1%) 48 (4.7%) 116 (11.4%) | 0.285 0.822  0.622 0.815 0.376 |
| Child gestational age (weeks) | 39.3 (2.0) | 39.4 (1.8) | 39.2 (2.0) | 0.944 |
| Child birthweight (g) | 3345.1 (554.6) | 3336.17 (520.2) | 3347.3 (563.2) | 0.420 |
| **STOP Follow-Up** |  | **Those who had 6M breastfeeding data (n=159)** | **Those who did not  (n=126)** | **p-value** |
| Age at STOP recruitment |  | 26.2 (5.1) | 25.8 (5) | 0.931 |
| BMI |  | 28.3 (7.7) | 27.2 (6.2) | **0.007** |
| SEI |  | 33.8 (14.7) | 33.6 (13.4) | 0.732 |
| Caucasian ethnicity |  | 141 (88.1) | 111 (88.1) | 0.448 |
| Education Status  Did not complete  Year 10 Year 12  Certificate  Bachelor  Higher degree |  | 1 (0.6%) 16 (10%) 39 (24.4%) 63 (39.4%) 34 (21.3%) 61 (5.6%) | 4 (3.2%) 17 (13.5%) 30 (23.8%) 43 (34.1%) 20 (15.9%) 7 (4.4%) | **0.017** |
| Pregnancy complication^ GDM Gestational hypertension Preeclampsia Preterm Birth Small for gestational age |  | 28 (17.5%)  12 (7.5%)  15 (9.4%)  7 (4.4%)  21 (13.1%) | 14 (11.%) 6 (4.8%)  13 (10.3%) 7 (5.6%) 17 (13.5%) | 0.239 0.612  0.923  0.894 0.686 |
| Child gestational age (weeks) |  | 39.4 (1.7) | 39.3 (1.9) | 0.455 |
| Child birthweight (g) |  | 3316.8 (533.1) | 3351.9 (522.1) | 0.737 |
| **STOP Follow-Up** |  | **Those who were compliant with blood and physiological test (n=103)** | **Those who were not  (n=187)** | **p-value** |
| Age at STOP recruitment |  | 26.6 (5.5) | 25.7 (4.8) | 0.099 |
| BMI |  | 29.3 (6.7) | 27.2 (7.2) | 0.985 |
| SEI |  | 34.1 (14.5) | 34.2 (14.6) | 0.565 |
| Caucasian ethnicity |  | 90 (87.4%) | 167 (89.3%) | 0.459 |
| Education Status  Did not complete  Year 10 Year 12  Certificate  Bachelor  Higher degree |  | 2 (1.9%) 5 (4.9%) 26 (25.2%) 39 (37.9%)  21 (20.4)  10 (9.7%) | 3 (1.6%) 28 (15.8%) 45 (21.4%) 69 (36.9%) 32 (17.1%)  10 (5.3%) | **0.024** |
| Pregnancy complication^ GDM Gestational hypertension Preeclampsia Preterm Birth Small for gestational age |  | 19 (10.2%)  7 (6.8%)  15 (8%)  12 (6.4%)  22 (11.8%) | 21 (20.4%)  11 (5.9%)  14 (13%)  2 (1.9%)  15 (14.6%) | 0.070  0.863  0.269  0.232  0.668 |
| Child gestational age (weeks) |  | 39.6 (1.6) | 39 (1.8) | 0.191 |
| Child birthweight (g) |  | 3348.1 (466.6) | 3343.1 (542.4) | 0.285 |

*differences between groups
^ pregnancy complications are not mutually exclusive, one woman may have multiple pregnancy complications.
